# International Day of Radiology: Breast Imaging

**DOI:** 10.5334/jbr-btr.1232

**Published:** 2016-11-19

**Authors:** Chantal Van Ongeval, Julie Soens, Mireille Van Goethem

**Affiliations:** 1 University Hospitals Leuven, BE; 2 University Hospitals Antwerp, BE

**Keywords:** breast imaging, mammography, magnetic resonance imaging, breast ultrasound

## Abstract

November 8, 2016, is the International Day of Radiology (IDoR), which is dedicated to breast imaging and the essential role that radiology plays in the detection, diagnosis, and management of diseases of the breast (http://www.internationaldayofradiology.com). On the website, you can find the book to honour the International Day of Radiology, Screening & Beyond, which provides an amazing overview of breast imaging, with contributions from many of the world’s top breast radiologists.

Breast cancer is the most common cancer in women worldwide, with nearly 1.7 million new cases diagnosed in 2012. Belgium had the highest rate of breast cancer (age-standardized rate per 100,000 is 111.9), followed by Denmark (105 per 100,000), and the Netherlands (99 per 100,000). Nevertheless, Belgium had the highest proportion of breast cancer survivors alive five years after their diagnosis [1].

Already in the 1950s, radiologists pointed out the value of mammography in the diagnosis of breast disease. Professor Charles Gros of Strasbourg was one of the pioneers in Europe: in 1965, Charles Gros developed and tested the prototype Senographe in cooperation with the CGR Company (Compagnie Générale de Radiologie, France). This Senographe was the first dedicated mammography unit, manufactured strictly for mammography [2]. The real “mammogram” was born with a heightened contrast between breast tissue and fat and better resolution; therefore, masses and calcifications were better visualized. Gros also emphasized the potential of mammography in the detection of occult cancer, and in the 1970s, screening programs with mammography were set up [2].

During the following 30 years, conventional film-screen mammography (FSM) has been the method of choice for the radiological evaluation of the breast. Specific film-screen combinations and continuously improved film processing provided high-quality images. This improvement in technology and image quality resulted in a better detection of small, non-palpable cancers. With the introduction of organized breast cancer screening in some countries in Europe, a protocol for physical-technical quality control was developed to guarantee the best image quality at the lowest possible dose, with a low recall rate and high cancer detection rate. However, in most countries, this physical-technical quality control was only required for mammography systems participating in a screening program. The first edition of the document *European Guidelines for Quality Assurance in Mammography Screening* was an initiative within the Europe Against Cancer Programme, followed by the *European Guidelines for Quality Assurance in Breast Cancer Screening and Diagnosis*, fourth edition, which also included the quality control of the diagnosis of breast cancer [3,4]. Now the European Initiative on Breast Cancer (ECIBC) is working on a project to develop a European quality-assurance scheme for breast cancer services [5].

Since 1989, several loco-regional initiatives have developed pilot screening programs in Belgium. In as early as 1995, Leuven and Brussels were members of the European Breast Cancer Network (EBCN). Moreover, in the early 1990s, the government ordered the creation of pilot projects, and Gent, Antwerp, Brussels, and Leuven started different projects, inviting women from 50 to 70 years old to have a mammogram every 2 years. In 2001, screening with FSM was started in Belgium using a decentralized model. In 2005, the use of FFDM has been allowed in the screening flowing a strict regulation, based on the chapter on digital mammography in the *European Guidelines for Quality Assurance in Breast Imaging*.

To date, the role of mammography in reducing breast cancer mortality has been demonstrated in multiple randomized clinical trials as well as in organized mammography screening services, of which Belgium is one. According to the International Agency for Research on Cancer, the reduction in mortality is 40 percent for women 50–69 who take up the invitation for screening mammography (position paper on screening for breast cancer by the European Society of Breast Imaging (EUSOBI) and 29 national breast radiology bodies). Parallel to this continuous improvement of mammography, new imaging techniques were introduced.

Ultrasound of the breast is now the standard of care for the evaluation of palpable lesions and of the additional evaluation of dense breasts. Different studies proved a higher cancer detection rate, but at the price of a lower specificity with an increase of minimal invasive procedures for benign structures. With higher quality of the ultrasound systems and the introduction of elastography, characterization of the lesions improved with an impact on the biopsy procedures. One of the main advantages of ultrasound is the use as a guidance tool for cytological and histological sampling of breast lesions. As fine needle aspiration was widely used in the 1980s, core needle biopsy (CNB) became the standard care technique in the evaluation of suspicious breast lesions. Because CNB of microcalcifications led more frequently to an underestimation of histological diagnosis, the vacuum-assisted biopsy (VAB) was introduced and is now widely used in all lesions that need large samples. Nowadays, pre-operative imaging and CNB/VAB are quality parameters in the evaluation of breast clinics.

In the early 1990s, the first magnetic resonance imaging (MRI) of the breast was performed. Introduction of kinetic MRI with gadolinium-DTPA resulted in differentiating benign from malignant lesions with a high sensitivity of up to 98 percent of invasive breast cancer and a gradually improved specificity. MRI has a level 1 evidence for the pre-operative evaluation of multifocal breast cancer and has proven to find smaller cancer in women with a strong family history of breast cancer and gene mutation carriers [6].

New research on the use of X-ray in digital breast imaging resulted in the development of digital breast tomosynthesis (DBT) and contrast-enhanced mammography (CEM). Clinical trials, not only regarding the value in diagnostic imaging but for DBT, in screening settings are ongoing [7].

The detection and diagnosis of breast cancer has a large impact on the lives of women; the different imaging techniques need to be of high quality and must result in an accurate detection and diagnosis, with high sensitivity and specificity. This implies the requirement that all mammography systems in Belgium should have a physical-technical evaluation according to the European guidelines. Integrating the results of the different technique in one comprehensive report is a goal for all radiologists performing breast imaging.

Throughout the development of different breast-imaging techniques, high image quality and quality control have been the common thread throughout the story of screening for breast cancer, but also for the diagnosis and treatment of breast cancer. An improved use of recent techniques must result in higher sensitivity and specificity at the best possible comfort of the women while offering an even better prognosis.
